# Synthesis of molecularly imprinted polymer for removal of Congo red

**DOI:** 10.1186/s13065-020-00680-8

**Published:** 2020-04-03

**Authors:** Syed Rizwan Shafqat, Showkat Ahmad Bhawani, Salma Bakhtiar, Mohamad Nasir Mohamad Ibrahim

**Affiliations:** 1 Faculty of Resource Science and Technology, Universiti Malaysia Sarawak (UNIMAS), 94300 Kota Samarahan, Sarawak Malaysia; 2 School of Chemical Sciences, Universiti Sains Malaysia, 11800 Gelugor, Pulau Penang Malaysia

**Keywords:** Congo red, Synthesis, Molecularly imprinted polymer, Removal, River water

## Abstract

Congo red (CR) is an anionic azo dye widely used in many industries including pharmaceutical, textile, food and paint industries. The disposal of huge amount of CR into the various streams of water has posed a great threat to both human and aquatic life. Therefore, it has become an important aspect of industries to remove CR from different water sources. Molecular imprinting technology is a very slective method to remove various target pollutant from environment. In this study a precipitation polymerization was employed for the effective and selective removal of CR from contaminated aqueous media. A series of congo red molecularly imprinted polymers (CR-MIPs) of uniform size and shape was developed by changing the mole ratio of the components. The optimum ratio (0.1:4: 20, template, functional monomer and cross-linking monomer respectively) for CR1-MIP from synthesized polymers was able to rebind about 99.63% of CR at the optimum conditions of adsorption parameters (contact time 210 min, polymer dosage 0.5 g, concentration 20 ppm and pH 7). The synthesized polymers were characterized by various techniques such as Fourier Infra-red spectroscopy (FTIR), scanning electron microscopy (SEM), Thermogravimetric analysis (TGA), energy-dispersive X-ray spectroscopy (EDX), and Brumauer–Emmett–Teller (BET). The polymer particles have successfully removed CR from different aqueous media with an efficiency of about ~ 90%.

## Introduction

Dyes are important ingredients in many industries such as textile, paints, cosmetics, pharmaceuticals, papers and printing [1]. They are capable of causing various harmful effects (toxicity, carcinogenic and mutagenic) on both human and aquatic life [2]. The colored effluent can reduce the oxygen levels in water and can affect photosynthetic processes of aquatic plants that resulted in the damage of aquatic flora and fauna [3, 4]. Congo red (CR) is an anionic dye and is widely being used in many industries. The elimination of CR from polluted water is important because it can be metabolized to carcinogenic agents (benzidine derivatives) if persisted. Therefore, different physical and chemical methods have been used for the removal of dyes from effluents such as chemical precipitation [5], chemical oxidation [6], adsorption [7], microbial or enzymatic treatment [8], and photo catalysis [9], mineral composites [10], natural sorbent materials [11, 12].

The need of more specific method for the removal of Congo red from different sources of water is essential. Therefore, molecularly imprinted polymers are the right choice for the removal of various pollutants from water effluents because of selectivity and specificity of method. MIPs are synthetic highly cross-linked materials with high ability to recognize and bind target molecules with specificity and affinity comparable to those of natural receptors [13–15]. Because of many advantages of MIPs such as specific recognition, chemical stability, and comparatively cheap and easy preparation [16] they have been widely employed for a variety of applications such as separation [17] solid-phase extraction (SPE) [18] sensors [19].

The main aim of this work was to synthesize the molecularly imprinted polymers for the selective removal of Congo red from different water environments. In this study a new porogenic solvent was used in form of mixture of dimethyl sulfoxide and acetonitrile. In the preparation of MIPs different approaches have been used to establish an interaction between template and monomer. But, in this study a non-covalent approach has been employed to produce MIPs. In this work methacrylic acid has been used as a monomer to establish a hydrogen bonding with the Congo red within the imprinted polymer.

## Materials and methods

### Materials

Congo red (CR) and methyl yellow (MY) were obtained from Bendosen laboratory chemicals, Malaysia. Methacrylic acid (MAA), ethylene glycol dimethyl acrylate (EGDMA), and 2, 2-azobisisobutyronitrile (AIBN) were supplied by Sigma-Aldrich, Germany. Acetic acid, acetone, acetonitrile (ACN), dimethyl sulphoxide (DMSO) and methanol, were purchased from R&M Chemicals, UK. All chemicals were used as obtained.

### Instruments/equipments

FT-IR spectra of the MIP and the NIP were recorded by FT-IR analysis. KBr disks of the MIP and the NIP were respectively prepared, and their spectra were recorded at 4000–400 cm^−1^ (Model ThermoScientificNicoletiS10). Electron micrographs were taken for evaluation of the morphology with a scanning electron microscope (SEM) (Model JEOL JSM-6390LA). All the samples were analyzed on a stub of aluminum coated under vacuum with a thin layer (40–60 nm) of gold. The thermal analysis of the molecular imprinted polymers was carried out by using TGA Instrument, Universal Analyzer 2000 with Universal V4.7A software. The sample was analyzed over a temperature range of 30–900 °C at a heating rate of 10 °C/min. The surface area and pore size were determined at 77 K by N_2_ adsorption and desorption isotherms using multipoint Brumauer–Emmett–Teller (BET) method. The other equipment’s used in this research were bath sonicator (Model Branson 2510), centrifuge (Model Hettich EBA20), shaker (Model N-Biotek 101MT) and water bath (Model Memmert W350T).

### Procedure for the synthesis of molecular imprinted polymers (CR1-MIP, CR2-MIP, CR3-MIP& CR1-NIP)

In this synthesis, 0.1 mmol of Congo red (CR) as a template was added in a 250 mL conical flask labeled “1” containing 10 mL of Dimethyl sulphoxide (DMSO); 65 mL Acetonitrile (ACN) as a porogen. Since the solubility of Congo red in Acetonitrile is very low so a mixture of ACN/DMSO had been employed. Dye was allowed to dissolve completely in porogen by sonication and then 4.00 mmol of Methacrylic acid (MAA) was taken as functional monomer in the conical flask followed by the addition of 20.00 mmol of Ethylene glycol dimethyl acrylate (EGDMA) as a cross-linking monomer. To initiate the free radical polymerization process, 0.030 g of 2, 2-azobisisobutyronitrile (AIBN) as a polymerization initiator was added into the mixture. Hence, the molar ratio of template: monomer: cross-linker for CR1-MIP was 0.1:4:20, respectively. The aggregate of this ratio was dissolved by using sonicator for 10 min to get well homogenized charge. After that, the mixture was purged with nitrogen gas (N2) for 15 min. The conical flask containing mixture was sealed and contents were allowed to polymerize in water bath at 60 °C for the first 6-h followed by 80 °C for the next 2-h. The developed polymer particles were then filtered by using vacuum filtration assembly. Same procedure and protocol was adopted for the synthesis of two other ratios as listed in Table 1. As a control, NIPs synthesis had been conducted on the same parameters and procedures as for CR-MIPs ratios but template molecule (Congo red) was omitted from the polymerization process.

**Table 1:** Composition of CR-MIPs/NIP

Polymers	Template (mmol/L) Congo red	Functional ionomer (MAA) (mmol)	Cross linking ionomer (EGDMA) (mmol)	Polymerization initiator (AIBN) (mmol)	Porogenic solvent
CR1-MIP	0.1 mmol	4.00	20.00	0.1	DMSO (10 mL) ACN (65 mL)
CR2-MIP	0.1 mmol	6.00	20.00	0.1	DMSO (10 mL) ACN (65 mL)
CR3-MIP	0.1 mmol	8.00	20.00	0.1 mmol	DMSO (10 mL) ACN (65 mL)
NIP-1	NA	4.00	20.00	0.1 mmol	DMSO (10 mL) ACN (65 mL)

### Washing of CR-MIPs

The synthesized polymer microspheres were washed with MeOH/Acetic acid mixture (9:1, v/v, of Methanol and Acetic acid) by an orbital shaker for 15 min at 250 rpm and then were filtered to get template free MIPs. As Methanol is protic in nature so it can easily crack the H-bonding established between Congo red and the polymer. So, same procedure (washing and filtration) was repeated until Congo red cannot be detected in liquid by UV–Vis spectrometer. Lastly, the polymer microspheres were washed with pure Methanol to omit Acetic acid and dried for 24 h in an open air [20]. All the completely washed and dried MIPs were collected in glass veils labeled as “after wash”.

### Batch binding assay (rebinding assay)

Three different conical flasks (250 mL) containing 20 ppm of standard Congo red solution were added with 0.5 g of CR1-MIP, CR2-MIP and CR3-MIP polymer samples respectively. All these assemblies containing polymer were agitated on an orbital shaker at 150 rpm for 6 h continuously. The samples were collected into glass vials at every 30-min time interval. The collected samples were then centrifuged at 4000 rpm for 10 min. Batch binding for NIP was carried out using the same procedure as followed for CR-MIPs.

Removal efficiency of Congo red dye by the CR-MIPs and NIP was evaluated by UV–Vis spectrophotometer which leads to measure the extraction efficiency of CR-MIPs and NIP. The λmax of standard CR solutions was secured at 497 nm. So absorbance of each extracted sample was determined at λmax.

Following mathematical equation was used to calculate the extraction efficiency (Q) of the samples:

1 $$ {\text{Extraction efficiency Q }}\left( \% \right) = \frac{Co - Cf}{Co} \times 100 $$

where, Co is defined as initial concentration (mg/L) of CR in solution; Cf is defined as final concentration (mg/L) of CR in solution.

Results obtained from the UV–Vis spectroscopy and then formulated extraction efficiency/absorbance capacity data justified that considerable amount of Congo red dye was adsorbed/extracted by CR1-MIP during batch binding of all the MIPs synthesized in this task. Thus, CR1-MIP with molar ratio of 0.1:4:20 for template: functional monomer: cross-linking monomer respectively was selected for further studies and investigations.

### Regeneration of CR1-MIP

Adsorbed Congo red dye by the CR1-MIP during batch binding procedure was then desorbed by washing with a mixture of MeOH/Acetic acid mixture (8:2, v/v) for further use. The washing procedure was repeated until to make sure that the entire template had been removed. Washing with a mixture of Methanol: Acetic acid (8:2, v/v) for further use. The washing procedure was repeated many times to make sure the entire template had been removed.

### Effect of CR dye concentration on its uptake behavior by CR1-MIP

Different concentrations (10 ppm, 15 ppm, 20 ppm, 25 ppm and 30 ppm) of Congo red were prepared for the evaluation of both of the selected polymers in this study. A constant contact time and dosage of the polymer were subjected for optimizing the optimum concentration. A series of conical flasks was used and labelled as 1, 2, 3, 4, and 5 for the study of the effect of initial dye concentration on CR1-MIP. All the flasks (1, 2, 3, 4 and 5) containing 0.5 g of CR1-MIP were added with different concentrations of Congo red (10 ppm, 15 ppm, 20 ppm, 25 ppm and 30 ppm respectively). All the flasks were kept on an orbital shaker and were agitated at 150 rpm. After the optimum time interval, the samples were collected and filtered for the removal of molecular imprinted polymers. The filtrates were then subjected to UV–Vis spectrometric analysis for the concentration determination of Congo red at λmax 497 nm.

### Effect of CR1-MIP dosage on its uptake behavior of CR

In this study, a series of conical flasks was used and labelled as 1, 2, 3, 4 and 5 for different dosage of polymer (CR1-MIP). All the flasks (1, 2, 3, 4 and 5) containing optimum concentration of Congo red solution were added with different dosage of polymer CR1-MIP (0.1 g, 0.3 g, 0.5 g, 0.7 g and 1.0 g). All the flasks were kept on an orbital shaker and were agitated at 150 rpm up to their optimum contact time. The contents were then filtered for the removal of molecular imprinted polymers. The filtrates were then subjected to UV–Vis spectrometric analysis for the concentration determination of Congo red at λmax 497 nm.The removal efficiency for all the samples were calculated by applying Eq. 1.

### Effect of Congo red solution pH on its uptake behavior by CR1-MIP

The chemistry of dye molecules and the configuration of sorbent/adsorbent are obviously affected by the pH of aqueous media [21, 22], so this effect was monitored for Congo red uptake by CR1-MIP. In this study, the contact time, concentration of dye solution and dosage of the CR1-MIP were kept constant while pH of Congo red solution was changed. The pH of Congo red solution was adjusted in both acidic and basic range such as 5, 6, 7, 8 and 9 pH. The selected polymers CR1-MIP was analyzed in all of the adjusted pH values. A series of conical flasks was used in which the selected CR1-MIP was tested. A 0.5 g of CR1-MIP was added into set of conical flasks. The flasks were then subjected to orbital shaker for agitation at 150 rpm up to an optimum time interval. After due time period the flasks were taken out from shaker and the contents were filtered for the removal of molecular imprinted polymer. The filtrates were then subjected to UV–Vis spectrometric analysis for the concentration determination of Congo red at λmax 497 nm. The removal efficiency and for all the samples was calculated by applying Eq. 1 hence, optimum pH for CR1-MIP was recorded.

### Imprinting factor

Imprinting factor is a measure of the strength of interaction of the imprinted polymer towards the template molecule. It sounds the specific recognition properties of molecular imprinted polymer and non-imprinted polymer with respect to a specific template. The imprinting factor (IF) of molecularly imprinted polymer is defined by the following equation [23]:

2 $$ {\text{IF}}\left( \alpha \right) = \frac{{{\text{Q }}_{\text{MIP}} }}{{{\text{Q }}_{\text{NIP}} }} $$

where, Q_MIP_ = Adsorption capacity of CR1-MIP for Congo red; Q_NIP_ = Adsorption capacity of NIP for Congo red.

Imprinting factor for optimized CR1-MIP and its NIP was calculated from the batch binding assay data.

### Repeated use of optimized CR-MIPs

The repeated use of the sorbent (CR1-MIP), which is a strategic factor in improving the wastewater process economics, was valued in ten sequential cycles of Congo red dye adsorption–desorption. Loss in Congo red rebinding between 1st and 10th cycle was observed under optimum conditions of agitation time, dosage, concentration and pH of solution. The estimated very slight change in adsorption–desorption values of polymer (CR1-MIP) would give an evidence that the synthesized materials can be used repeatedly without losing considerably their adsorption capacities.

### Selectivity test for CR1-MIP

Ability of any sorbent/adsorbent material to differentiate and quantify its analyte in the intrusion of any other interferent in its matrix is known as selectivity [24]. The selectivity can be customized by enhancing the specific interactions (SI) situated inside imprints while minimizing the non-specific interactions (NSI) with the polymer stuff. Both CR1-MIP along with NIP were subjected to selectivity test. In order to perform this selectivity trial, 80 mL of 20 ppm binary solution was prepared by mixing 40 mL of standard 10 ppm Congo red solution with 40 mL of 10 ppm standard solution having a competitive template (Methyl yellow). The optimum contact time, optimum dosage of MIP/NIP and optimum pH were used for this study. All the flasks containing mixed solution of Congo red and Methyl yellow were agitated on an orbital shaker at 150 rpm. All the flasks were removed from the shaker after their optimum contact time period and the contents were filtered for the removal of polymers. The filtrates and standard solutions were then subjected to UV–Vis spectrometric analysis for the concentration determination of Congo red at λmax 497 nm and Methyl yellow at λmax 410 nm.

The distribution ratio (K_D_) of Congo red between the MIP or NIP with the porogen was determined by following the equation as:

3 $$ {\text{Distribution ratio}},{\text{ K}}_{\text{D}} = \frac{{\left( {{\text{C}}_{\text{i}} - {\text{C}}_{\text{f}} } \right){\text{V }}}}{{{\text{C}}_{\text{f }} {\text{m }}}} $$

where, C_i_: The initial dye (Congo red or Methyl yellow) concentration in solution; C_f_: The final (Congo red or Methyl yellow) concentration in solution; V: The volume of porogen used; m: The mass of MIP/NIP used.

The selectivity coefficient for Congo red relative to binding competitor Methyl yellow for MIP and NIP was defined as:

4 $$ {\text{K}}^{\text{sel}} = \frac{{{\text{K}}_{\text{D}}   {\text{Template }}\left( {\text{Congo red }} \right)  }}{{{\text{K}}_{\text{D}}   {\text{Interferent }}\left( {\text{Methyl yellow}} \right)}} $$

where, K_D_ Template: The batch binding assay of MIP/NIP for Congo red; K_D_ Interferent: The batch bindi assay of MIP/NIP for Methyl yellow.

Hence, the relative selectivity coefficient (K^′^) was determined by following equation as:

5 $$ \begin{aligned}{ {\text{K}}^{\prime}} = \frac{{{\text{K}}^{\text{sel}} \left( {{\text{CR}} - {\text{MIP}}} \right)}}{{{\text{K}}^{\text{sel}} \left( {\text{NIP}} \right)}}\end{aligned} $$

`

Similarly, selectivity factor for (β) for optimized CR1-MIP was also evaluated by applying following equation:

6 $$ \beta = \frac{{{\alpha}_{\text{template}} }}{{{{\alpha  }}_{\text{interferent }} }} $$

where, α_template_: Imprinting factor towards Congo red; α_interferent_: Imprinting factor towards Methyl yellow.

### Applications of Congo red MIPs in different water environments

#### Preparation of water samples

Finally, the successive confirmation of CR1-MIP produced was evaluated through rebinding process from water samples (Double distilled water, Tap water and River water) by using the similar method from batch binding assay. The water samples (Tap water/River water) were filtered and centrifuged to remove sediments prior any extraction and then stored in lab for time being. The optimized adsorption protocol was applied to water samples in order to evaluate the ability of selected CR1-MIP to specifically extract Congo red. For this study, a series of flasks was used and labelled as 1, 2, 3, 4, 4 and 6. These flasks were then divided into three different sets. The DDW (Double distilled water) spiked with Congo red was added into the first set of flasks (1, and 2) and similarly, the Congo red spiked in Tap water and River water were added in the second (3 and 4) and third set (5 and 6) of flasks respectively. After that, the optimized conditions such as optimum contact time, optimum dosage, optimum concentration and optimum pH were used for the study. The absorbance of Congo red by polymer particles from Congo red spiked water samples was examined in the extracted samples by UV–Vis spectrophotometer at λmax 497 nm. The removal efficiency for all the samples was calculated by applying Eq. 1 and hence, successive synthesis of molecular imprinted polymers for the extraction of azo dyes (Cong red) from water environments was confirmed.

## Results and discussion

### Synthesis of molecular imprinted polymers for CR

**Fig. 1:**
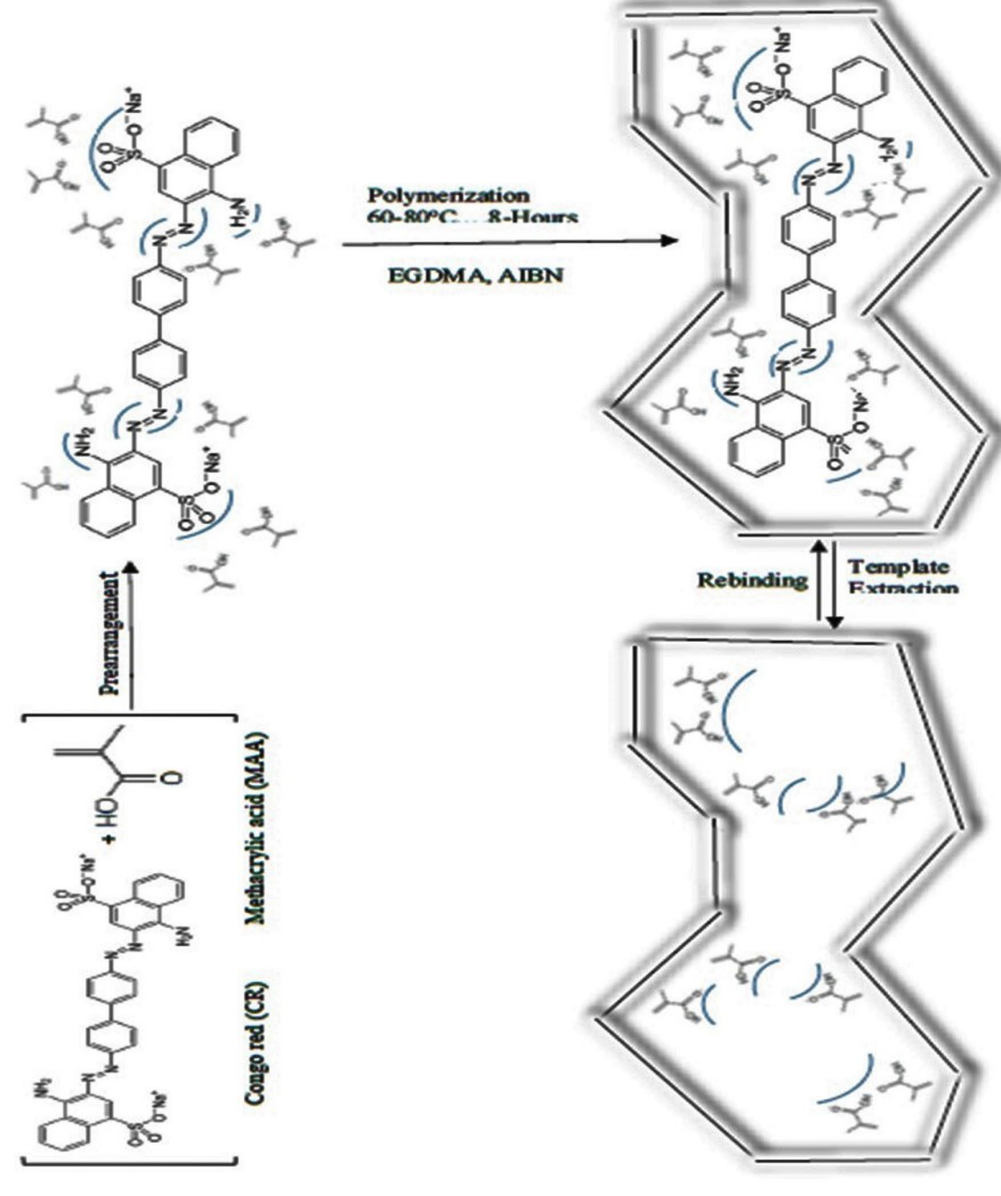
Schematic representation for the synthesis of MIP with CR as a template

A very large or very small selected template molecules can reduce imprinting effect so average sized Congo red dye molecule was employed as a template to produce MIPs bearing good imprinting effect. For the increase of affinity of template to exact fit into recognition sites, shape selection was also vital as the imprinted cavity must have similar shape as the template to show optimal imprinting effect. So, non-covalent approach was used to synthesize MIP particles imprinted with well orientated azo dye molecules. A schematic representation for the synthesis of CR-MIPs is illustrated in Fig. 1. In molecular imprinting, weak interactions (electrostatic interactions and hydrophobic interactions etc.) may contribute to the interaction between the template molecule and the functional monomer in porogen prior to polymerization. Determining the best choice of the functional monomer and the absolute proportions of the polymerization reagents can assist in creating a significant imprinting and extraction performance. Since this will influence the stability of the framed polymer before and during the polymerization process and thus the execution of the MIPs to interface particularly with the objective analyte [25]. So, Congo red MIPs were synthesized successfully from co-polymerization of Methacrylic acid (MAA). In the present study, H-bonding (non-covalent bond) was the dominant driving force for the molecular recognition between the functional monomer (carboxyl group of MAA in this case) and the target molecule (amine group/sulfonic group of Congo red in this case). So, carboxyl group of Methacrylic acid (Acidic functional monomer) facilitated it to form H-bonding with the amino groups (–NH_2_) and sulfonic groups (–SO_3_H) of Congo red molecule (Anionic dye). There are also a number of other possible sites available for specific and positioned interactions in template molecule (azo group; N=N and H–C-sites of aromatic rings) that may contribute to the MIP’s selective affinity. Crosslinking monomer can execute some important roles such as providing mechanical stability to the polymer matrix, regulate the morphology of the polymer and maintain the molecular recognition binding sites. EGDMA was chosen for this purpose. Hence, as long as the temperature of reaction was varied, productive generation of free radicals of the AIBN resulted in the start of reaction. So, upon Crosslinking by EGDMA, a strong backbone of the polymer was formed where the Congo red dye molecules were entrapped. By leaching of the Congo red dye molecules from the polymer matrix, a molecularly imprinted polymer material was generated where recognition sites were composed of the functional groups and the shape complementary to the structure of Congo red dye molecules.

### Batch binding assay for CR-MIPs

**Fig. 2:**
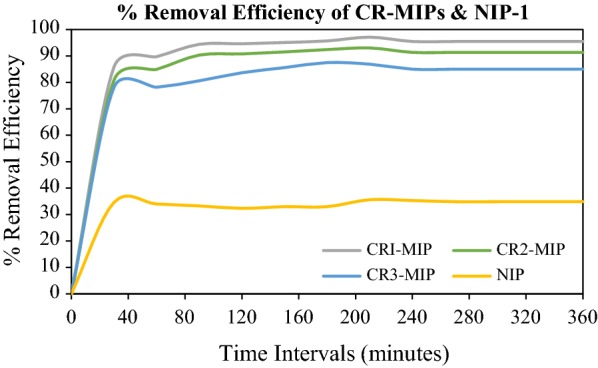
Batch binding analysis of CR-MIPs

Batch binding technique was utilized to study the rebinding adsorption, removal efficiency and binding affinity/capacity [26] of molecular imprinted polymers of Congo red dye. It also helped to investigate the highly selective MIP for target analyte out of a series of compositions. So, a better MIP with greater number of binding sites was obtained through batch binding assay. Batch binding was conducted to assess the magnitude of molecular recognition of developed MIPs and this molecular recognition depends upon two factors mainly, three-dimensional spatial configuration of the template molecule and the matching degree of the bonding sites. Figure 2 portray that amongst the three different ratios of the synthetic series of CR-MIPs, CR1-MIP (0.1:4:20) exhibited the highest removal efficiency of 97.12%. This higher efficiency of CR1-MIP was due to its compatible ratio of template, functional monomer and crosslinking monomer which may have the greater specific recognition sites for the Congo red as compared to the other two CR-MIP ratios. As the ratio of functional monomer to template is increased, the nonspecific affinity of imprinted polymer is also increased [27] and because of this effect CR2-MIP and CR3-MIP showed less binding affinity towards specific analyte (Congo red) and resulted in lower removal efficiency (Max. 93.04% and Max. 87.50% respectively). Addition of an excess amount of MAA both in CR2-MIP and CR3-MIP, perhaps prompt the self-association [28] of MAA molecules that leads to decrease in generation of binding sites on the respective MIP.

As shown in Fig. 2, the removal efficiency of the representative members of CR-MIPs series is increasing with the increase in contact time and reached a maximum at 210 min. Further increase in contact time did not produced any significant increase in the removal efficiency. This means that adsorption equilibrium has been achieved at 210 min. In fact, the binding sites were fully saturated and after that the template molecules were not able to implant in the binding sites of MIPs anymore beyond this equilibrium contact time. So, the highest efficiency of CRI-MIP was achieved at 210 min. The rebinding efficiency of corresponding non imprinted polymer (NIP-1) was very low and linear over the entire time scale. This may be due to the non-availability of complimentary binding sites.

### Effect of CR dye concentration on its uptake behavior by CR1-MIP

**Fig. 3:**
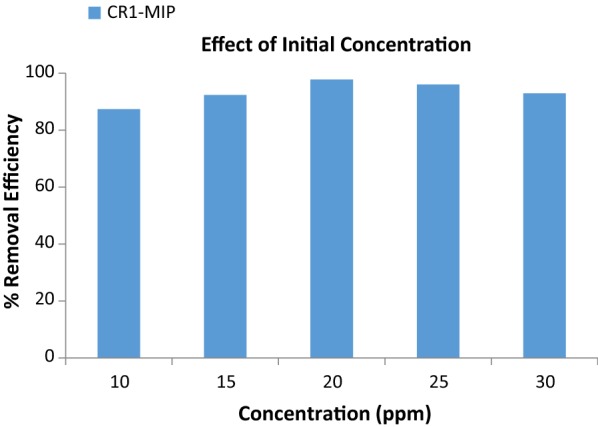
Effect of CR dye concentration on its uptake behavior by CR1-MIP

Initial dye concentration has a very outward impact on its removal from any aqueous phase by molecular imprinted polymers. The removal efficiency (% R) of CR1-MIP sounds to be increased with the increase in the initial dye concentration of Congo red (Fig. 3). By increasing the initial dye concentration, molecules of dye will be higher that in turn will surround the active binding sites of the polymer (CR1-MIP) effectively, leading to a better and efficient adsorption/sorption. This trend can also be ascribed by the fact that if dye concentration is higher then, responsible driving forces for mass transfer will be relatively high. The maximum removal efficiency for CR1-MIP was observed 97.12%, at an initial Congo red dye concentration of 20 ppm. After that no apparent change was found in dye uptake behavior at higher concentrations because all the binding sites have been saturated with the dye molecules. The further increase in dye concentration did not produced any significant increase in removal efficiency.

### Effect of CR1-MIP dosage on its uptake behavior of CR

**Fig. 4:**
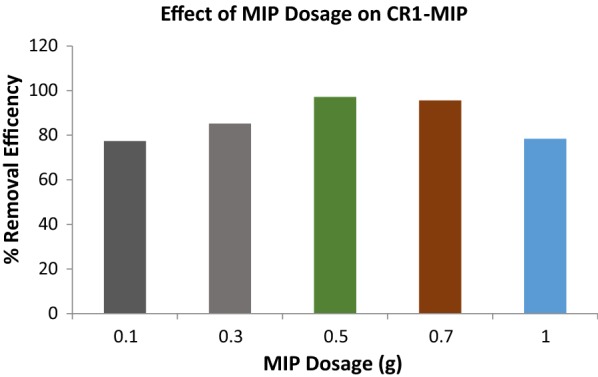
Effect of Congo red solution pH on its uptake behavior by CR1-MIP

The removal efficiency of dye increases with the increase in the adsorbent dosage up to a certain limit but after that there is no significant increase in the removal of dye but a gradual decline. Graphical representation (Fig. 4) can be justified by the fact that increase in polymer dosage, number of the adsorption sites were increased and dye molecules may spread on CR1-MIP that bring about increase in adsorption. But further increase in polymer dosage have decreased the removal efficiency of the imprinted polymer. This decrees in adsorption can be explained in the way that the increase in polymer dosage have aggregated the polymer particles. This aggregation (inter particle attraction) of polymer particles have decreased the available binding sites and hence less available binding sites attributed the decrease in removal efficiency of CR1-MIP by increasing the polymer dosage. The maximum removal efficiency (97.12%) was achieved at a polymer dosage of 0.5 g. So, dosage of 0.5 g was considered as optimum dosage for optimized CR1-MIP at optimum agitation time and optimum concentration.

### Effect of Congo red solution pH on its uptake behavior by CR1-MIP

**Fig. 5:**
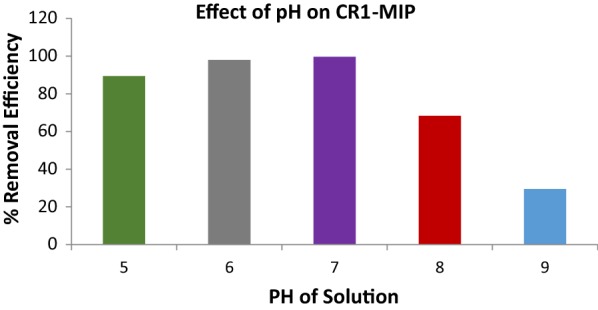
Effect of Congo red solution pH on its uptake behavior by CR1-MIP

Fabricated molecular imprinted polymers tend to uptake the target analyte at neutral pH but this phenomenon is absolutely dependent on the presence of acidic or basic functional groups of analyte and the nature of active functional sites present inside the cavities of MIPs [29]. Due to chemical speciation of Congo red dye in the pH range of 2–5 and 12 [30], the effect of pH of Congo red dye solution on the removal efficiency and adsorption by optimized molecular imprinted polymers was studied in the pH range of 5–9 at optimized dosage of MIP, contact time and concentration. The pH of medium has a significant effect on the adsorption of Congo red dye by MIP. This is due to the chemical speciation for both of the active binding sites of cavities present inside the MIP and the active functional groups of dye available for bonding. Any change in cavity or dye molecule’s configurations will mismatch for proper “fit into” leading to alter the adsorption capacity and removal efficiency. So, it is very important to evaluate the impact of initial pH of medium on adsorption. Figure 5 shows the effect of pH of solution on the removal efficiency of Congo red dye by CR1-MIP. It is evident from the Fig. 5 that highest removal efficiency of Congo red dye was achieved at pH 7 with an efficiency of 99.63%. At optimum pH (pH 7) there are no structural changes of dye and MIP configuration’s so, maximum adsorption of dye was observed at neutral pH and then changed as the medium turned to acidic or basic.

The higher adsorption by CR-MIP at neutral media (low pH = 7) could be attributed to the considerable interactions between the carboxylic (–COOH) functional groups present inside the binding cavities of CR1-MIP and amino groups/azo groups/sulfonate groups of dye molecules. On the other hand, at lower pH (acid media) or higher pH values (basic media), the adsorption seems to be declined. Congo red (adsorbate) is predominantly present in ionic form in aqueous media having two anionic sulfonate groups (–SO_3_^−^) and the H-bonding probably seems to be weaken between these groups and carboxyl functional groups (–COOH) of MAA, present in the frame work of polymer (adsorbent/sorbent) in acidic or basic media. If pH is decreased, there will be numerous competing H^+^ ions in acidic media that will be available for the protonation of carboxylic group and amino groups. Increase of hydrophobic interactions and weakening of H-bonding between Congo red and selective binding sites of MIPs may be the main reason in a slight decreasing the binding capacity in acidic media. Similarly, at higher pH values (basic media), the adsorption also seems to be declined which may be due to the presence of soluble hydroxyl groups and competing with the dye anions for binding sites. As, structural and surface properties of both, MIP and dye molecules are induced by altering the pH solution with very low pH (strong acidic media) or higher pH (strong basic media) and hence, best working range for current stuff was considered at neutral pH (pH 7). Moreover, trend for pH factor always depends upon the availability of functional groups i.e. either acidic or basic in character [31–33].

### Imprinting factor of optimized CR-MIPs

Adsorption capacities of the imprinted polymers and their respective non-imprinted polymers aided to determine the specific recognition properties of CR-MIPs with respect to their template (Congo red) in the form of imprinting factors. It is a measure of the strength of interactions of the imprinted polymers towards template molecule as well as a measure of magnitude of interaction between the template and its corresponding functional monomer [34]. A good imprinting factor was achieved for the CRI-MIP as reported in Table 2.

**Table 2:** Study of imprinting factor for optimized CR1-MIP

Imprinted polymer	Imprinting factor formulation	Results
CRI-MIP	${\text{IF }}\left( \alpha \right) \, = \frac{{{\text{Q CRI}} - {\text{MIP}}}}{{{\text{Q NIP}} - 1}}$	2.80

### Repeated use of optimized CR-MIPs

Regeneration property is one of the important advantages of the molecular imprinted polymers. MIPs can be employed again to adsorb the interested analyte after regeneration and maintained their adsorption capacity at an almost constant value.

Optimum conditions of contact time, dosage, concentration and pH were applied and adsorption–desorption cycle was repeated ten times. CR1-MIP was stable for the proceeding adsorption cycles without obvious decrease in the removal efficiency for water-soluble acid dye. Table 3 illustrated that loss in Congo red rebinding between 1st and 10th cycle was ~ 5% for CR1-MIP. This negligible loss in specific recognition and sorption for Congo red elucidates their excellent reuse/regeneration ability.

**Table 3:** Effect of reuse of CR1-MIP on the removal efficiency

Adsorption/desorption Cycle	Extraction efficiency For Congo red by CR1-MIP (%)
Cycle-1	99.63
Cycle-2	98.70
Cycle-3	98.23
Cycle-4	97.50
Cycle-5	97.18
Cycle-6	96.03
Cycle-7	95.12
Cycle-8	94.60
Cycle-9	94.50
Cycle-10	94.10
Overall loss after 10 cycles	5.56

### Selectivity test for optimized Congo red molecular imprinted polymers

Selectivity test was conducted to evaluate the sensing properties of molecular imprinted polymer for Congo red. Methyl yellow (MY) a competitive and structural analogous to template, exhibiting most of physical and chemical properties similar to Congo red was chosen as potential interfering compound.

The magnitude of the distribution values usually indicates the favorability of the adsorption process. A higher value of the distribution (K_D_) indicates favorable adsorption/sorption and the solute favorably migrates from the solution to the binding sites of the imprinted polymer. Findings in the Table 4 portray that distribution ratio of Congo red in CR1-MIP was obviously outstanding than the distribution ratios of its competitor (Methyl yellow). High values of selectivity coefficients indicate the imprinted polymer is very selective for Congo red dye removal and the imprinting process was successful. The selectivity of the CR1-MIP may also give some insights into the molecular recognition mechanism. This could be easily explained by the close homology of CR to MY, but there is still a difference between them, the spatial diameter of CR is larger than that of MY. There are two –SO_3_H functional groups and two –HN_2_ groups in CR, but there is only one –N(CH_3_)_2_ functional group in MY.

**Table 4:** The distribution ratios, selectivity coefficients, relative selectivity coefficients and selectivity factors for optimized CR1-MIPand NIP

Findings	Polymer	Analyte dye	
		Template-Congo red	Competitor-Methyl rellow
Distribution ratio K_D_	CR1-MIP	79	15.01
Distribution ratio K_D_	NIP-1	28	8.15
Selectivity coefficient K^sel^	CR1-MIP	5.27	0.189
Selectivity coefficient K^sel^	NIP-1	3.46	0.289
Relative selectivity coefficient K,	CR1-MIP	1.523	0.653
Selectivity factor *Β*	CR1-MIP	1.521	

This study also glorifies that –NH_2_ group (primary amine) and sulfonic groups of Congo red situated at the edges of molecule may have favored the interaction with the active binding sites of CR1-MIP and easily entrapped into the cavities rather than its competitor. Moreover, Congo red and Methyl yellow have their higher distributions in the CR1-MIP than NIP because NIP is lacking complementary binding sites. The higher distribution ratio of Congo red is because of the fact that CR1-MIP can recognize and bind the template molecules by specific binding sites that have been retained as a memory. Congo red can easily affix to the quite matched cavities in size and shape, while Methyl yellow can bind weakly because of nonspecific interactions [35].

### Fourier transform infrared spectroscopic (FTIR) analysis for CR-MIPs

**Fig. 6:**
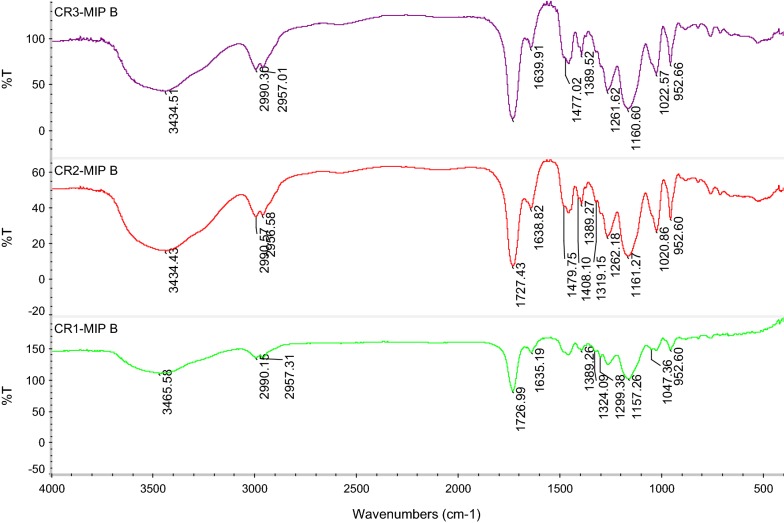
FTIR spectra of CR-MIPs

The wavelength of the absorption band in FTIR spectrum is the characteristics of chemical identity of the unknown sample and the intensity of the absorption band produces a quantitative analysis. The obtained FTIR spectra of CR-MIPs had a slight difference in the peak positions and their intensities. However, most of the position of characteristic peaks inspected in the absorption band spectra of CR-MIPs had similarities indicating the similarities in the backbone structure of the polymer [36]. In this study, FTIR analysis of EGDA cross linked poly (Methacrylic acid) CR-MIPs that have non-covalently entrapped the Congo red dye, have been observed and their secured spectra are shown in Fig. 6. In the spectra, the peaks at ~ 3430 cm^−1^ show the stretching vibration of N–H of Congo red. The peaks at ~ 2900 cm^−1^ are owing to the C-H stretching vibrations present in Congo red, EGDMA and Methacrylic acid. The strong peaks at ~ 1720 cm^−1^ reveal the presence of –COOH of Methacrylic acid. Moreover, the peaks ~ 1635 cm^−1^, ~ 1452 cm^−1^, ~ 1300 cm^−1^, and ~ 1200 cm^−1^ correspond to the presence of C = C, –CH_2_ stretching, –CH_3_ group and C–O stretching, respectively [37]. Furthermore, the band at ~ 1157 cm^−1^ associated to the sulfonate group of CR can be clearly seen suggesting the presence of CR in MIPs. Moreover, the peak at ~ 1023 cm^−1^ is very clear, which is another significant evidence of CR [38]. Hence, it is clear that Methacrylic acid successfully cross linked through EGDMA and Congo red dye is present in the CR-MIPs because the broad band around ~ 3465 cm^−1^ in the spectrum which is owing to the strong H-bonding between N–H groups of CR and –COOH group of Methacrylic acid. This broad band shows the presence of Congo red in cross linked Methacrylic acid network through physical interactions.

### EDX analysis of CR1-MIP

**Fig. 7:**
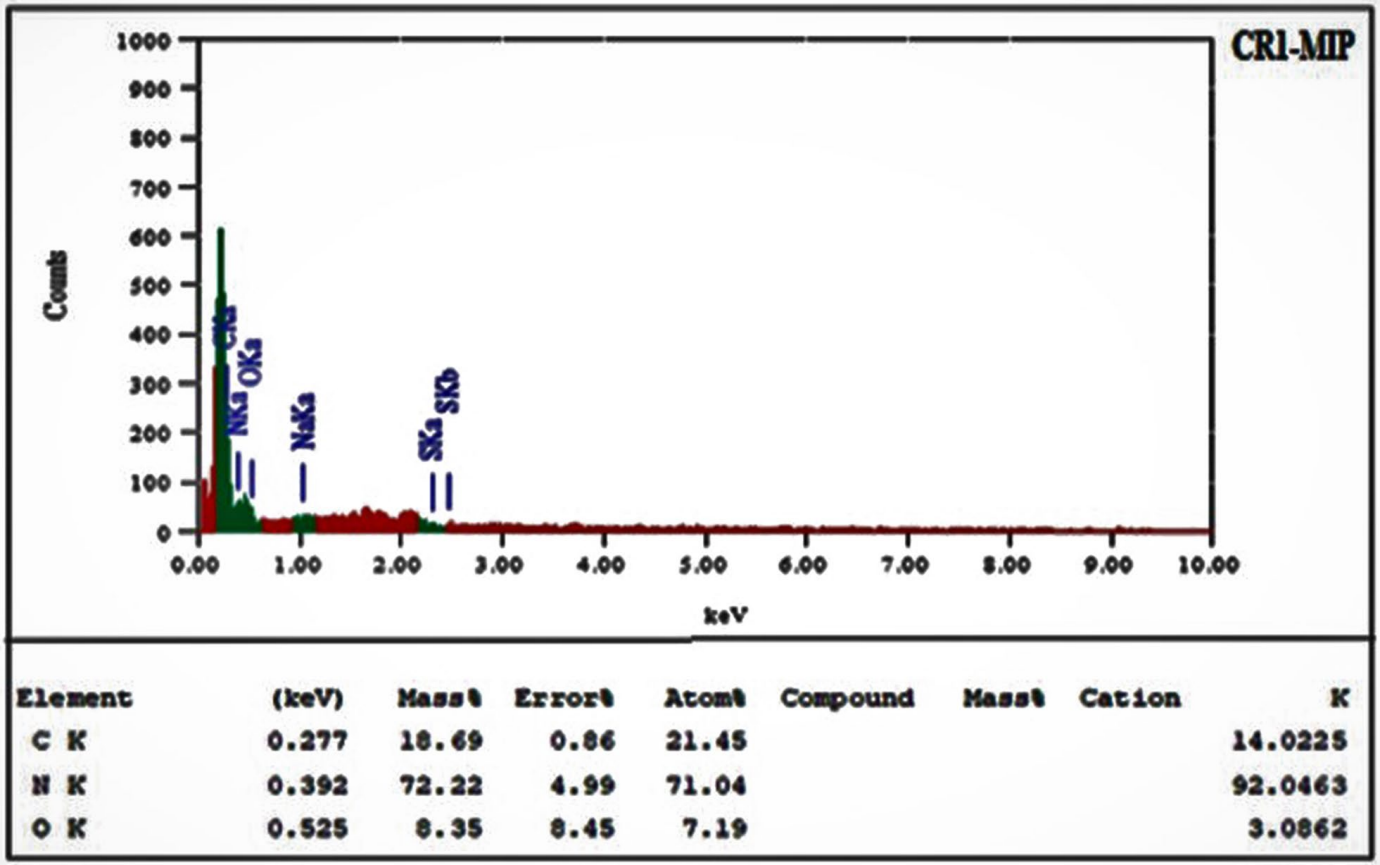
EDX Spectrum and elemental analysis chart for CR1-MIP

Synthesized CR1-MIP was analyzed by EDX immediately after the synthesis and drying without the leaching of template to evaluate the presence of each constituent of the participant in the polymerization process. Figure 7 shows the chemical composition of the polymer particles in the form of energy dispersive X-ray spectrograph for selected Congo red molecular imprinted polymer (CR1-MIP). The polymer particles are composed of C, N and O without the presence of any impurity. Spectrum reveals that the nitrogen was from Congo red, while carbon and oxygen were from MAA and EGDMA as well as from dye molecule too. The percent abundance of all components (quantitative analysis) in the backbone structure of Congo red imprinted polymer are depicted in the inset of Fig. 7.

### TGA analysis of CR1-MIP

**Fig. 8:**
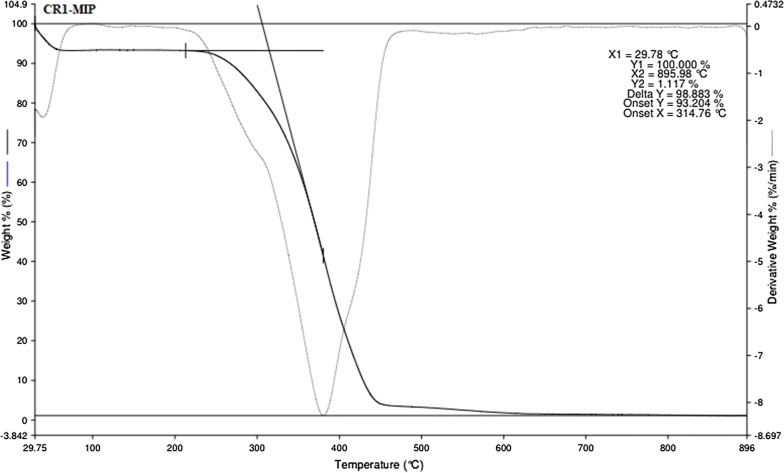
TGA spectrum of Congo red molecular imprinted polymerCR1-MIP

The thermal degradation behavior of EGDA cross linked poly (Methacrylic acid) CR1-MIP bearing binding cavities for Congo red dye was studied in the range of 30–900 °C. Figure 8 shows two stages of weight loss for CR1-MIP. Almost 7% weight loss was observed at first stage which showed the dehydration of water residues from the imprinted polymer. The second stage of high weight loss occurred in the range of 260–520 °C. This 90% weight loss of CR1-MIP might be owing to the decomposition of poly (Methacrylic acid) backbone. However, the literature reveals the complete degradation of pure Methacrylic acid around 100 °C, while this thermogram reveals the degradation of EGDA cross linked poly (Methacrylic acid) in the range of 260–520 °C [39]. This degradation at higher temperature shows the higher thermal stability of cross linked poly (Methacrylic acid) CR1-MIP as compared to pure Methacrylic acid.

Furthermore, DTA curves also showed two endothermic peaks which strongly support the phenomena of polymer degradation observed in TGA. In DTA curves, two endothermic curves can be seen. First endothermic curve is owing to the removal of moisture while second endothermic curve shows the decomposition of poly (Methacrylic acid) backbone. A peak on derivative curve of Poly-MAA with maximum decomposition rate at ~ 380 °C is due to CO_2_ release. The reported literature reveals the decomposition of poly (Methacrylic acid) in this range [40].

### Morphological study of CR1-MIP and NIP by using scanning electron microscope

SEM is an essential and valuable analysis technique for unfolding the understanding on the morphology and texture of the polymers. So, surface morphology of representative molecularly imprinted polymer CR1-MIP network and the respective control polymer (NIP) was assessed by scanning electron microscopy at 10,000 times magnifications over a bar scale of 1 µm, that provided an idea about the morphology of the polymer particles.

**Fig. 9:**
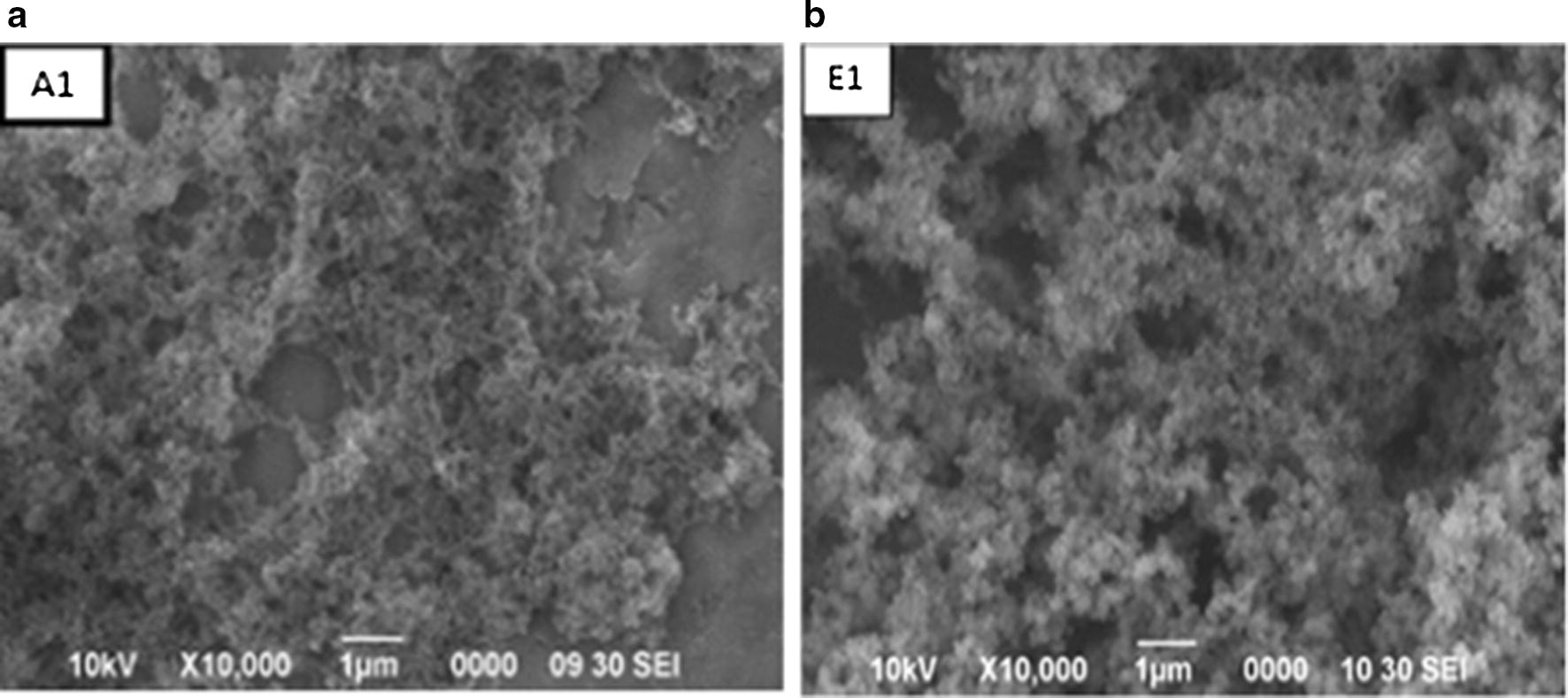
SEM of MIP (**a**) and (**b**) NIP

The micrographs in Fig. 9a and b, present the surface morphology of CR1-MIP particles The micrographs clearly revealed the surface with a porous texture having very tiny and homogeneous spherical particles that have been developed with a narrow size distribution in the range of micrometers ~ 0.07 µm. Uniformity in size and shape was owed to non-covalent precipitation polymerization method [41–44]. The uniformly sized MIP have advantages of possessing ‘large surface area, monodispersed, colloidal stability’ as compared to the irregularly shaped MIP particles. The uniform size of MIPs is the main reason to allow efficient template removal and fast binding kinetics because the binding sites are exposed to the surface [45] hence, better performance of CR1-MIP particles owed to their morphology.

For the development of of CR1-MIP, relatively a higher amount of acetonitrile was used as porogenic solvent. As the previous studies showed that SEM image analysis of MIPs prepared by acetonitrile exhibited a regular spherical shape [46].

### BET surface area analysis

BET analysis was performed to determine the specific surface area and porosity properties of the selected Congo red molecularly imprinted polymer i.e. CR1-MIP. Table 5 has been obtained and affords noteworthy surface areas, mean pore radius and pore volume of the MAA based CR1-MIP, as calculated from the adsorption isotherms. A noticeable and good porosity, indicating the polymer formed is porous in nature and this porosity is attributed to the fact that the use of solvent was porogenic [46, 47] and the evaporation of solvent led to the formation of pores within the CR1-MIP formed. In addition, the presence of cavities due to leaching of template molecule also contributed towards the porosity. It can also be perceived; higher surface area was obtained for CR1-MIP particles in comparison with NIP. These results showed that imprinting of Congo red have made the surface area larger and developed the porosity in CR1-MIP. The difference of adsorption efficiencies between the CR-MIP and NIP could not be attributed to the surface areas (morphological difference) only but also to the imprinting effect [48]. The mean pore radius of the CR-MIP was in the range of mesoporous particles [49]. A reasonable value of the data obtained proclaimed that the developed CR1-MIP was better sorbent, bearing three dimensional network complemented with Congo red in the polymers. Adsorption capacity and removal efficiency of CR1-MIP can be translated in the form of respective surface areas and total pore volume; higher they are, more they will imply working capacity [50].

**Table 5:** BET of CR1-MIP and CR1-NIP

Properties	CR1-MIP (magnitude)	CR1-NIP (magnitude)
Surface area (m^2^/g)	390	92.6
Average Pore Radius (Å)	8.881	1.374
Total Pore volume (cc/g)	1.735	0.344

### Applications of Congo red MIP in different water environments

The removal efficiency of CRI-MIP and NIP was evaluated to compare the potential interactions of imprinted and non-imprinted polymers towards water environments (Double distilled water, River water and Tap water) bearing Congo red as a pollutant. The results listed in Table 6 revealed that CR1-MIP exhibited higher removal efficiency of Congo red dye compared to NIP in any water environment. Removal efficiency of CR1-MIP seems to be slightly higher for Double distilled water environment rather than for Tap water and River water. Presence of organic/inorganic matter in natural water systems (Tap water/River water) may have interfered for selective uptake of dye molecules and became a cause of reduction in their selective removal efficiency. As metal ions (Ca^2+^ and Mg^2+^) are probably known to extant in River water and to establish coordinate covalent bonds with ligand possessing –COOH, –SO_3_H and –NH groups. It is assumed that may form coordinate bonds with the carboxyl and the amino moiety of Congo red dye and binding sites of polymer leading to decrease in binding characters. Hence, removal efficiency of CR1-MIP in River water environment is even less than that of Tap water environment.

**Table 6:** Removal efficiency of CR-MIP in different water environments

Samples	Amount of CR added (µg/mL)	CR1-MIP			CR1-NIP		
		Amount of CR found (µg/mL)	Recovery (%)	RSD (%)	Amount of CR found (µg/mL)	Recovery (%)	RSD (%)
Distilled water	20	19.42	97.12	0.31	7.12	35.61	0.54
Tap water	20	18.64	94.6	0.15	6.52	32.61	0.33
River water	20	18.02	90.12	0.85	6.32	31.61	0.90

## Conclusion

Precipitation polymerization of poly (MAA) CR-MIPs can be successfully employed for the effective and selective removal of CR from contaminated aqueous media. A series of CR-MIPs of uniform size and shape was developed by changing the mole ratio of the components. The optimum ratio (0.1:4: 20, template, functional monomer and cross-linking monomer respectively) for CR1-MIP from synthesized polymers was able to rebind about 99.63% of CR at the optimum conditions of adsorption parameters (contact time 210 min, polymer dosage 0.5 g, concentration 20 ppm and pH 7) as compared to other imprinted and non-imprinted polymers. These polymer particles have successfully extracted CR from different aqueous media. The extraction efficiency of selected CR1-MIP from CR spiked water samples was ~ 90%. The high imprinting factor of 2.80 for CR1-MIP is indicative of high selectivity of the adsorption for CR. The CR1-MIP was applied in ten sequential cycles of adsorption–desorption with a minimal loss of removal efficiency of only ~ 5.56%.

## Data Availability

All data is compiled in the manuscript.

## References

[1] Toor M, Jin B, Dai S, Vimonses V (2015). Activating natural bentonite as a cost-effective adsorbent for removal of Congo-red in wastewater. J Ind Eng Chem.

[2] Foroughi-dahr M, Abolghasemi H, Esmaieli M, Nazari G, Rasem B (2015). Experimental study on the adsorptive behavior of Congo red in cationic surfactant-modified tea waste. Process Saf Environ Prot.

[3] Cheung WH, Szeto YS, McKay G (2009). Enhancing the adsorption capacities of acid dyes by chitosan nano particles. Bioresour Technol.

[4] Hu TL, Wu SC (2001). Assessment of the effect of azo dye RP2B on the growth of a nitrogen fixing cyanobacterium–Anabaena sp. Bioresour Technol.

[5] Oladoja NA, Aliu YD, Ofomaja AE (2011). Evaluation of snail shell as a coagulant aid in the alum precipitation of aniline blue from aqueous solution. Environ Technol.

[6] Ersöz G, Napoleoni A, Atalay S (2013). Comparative study using chemical wet oxidation for removal of reactive Black 5 in the presence of activated carbon. J Environ Eng.

[7] Jia Z, Liu J, Wang Q, Li S, Qi Q, Zhu R (2015). Synthesis of 3D hierarchical porous iron oxides for adsorption of Congo red from dye wastewater. J Alloys Compd.

[8] Li X, Zhang J, Jiang Y, Hu M, Li S, Zhai Q (2013). Highly efficient biodecolorization/degradation of Congo red and Alizarin Yellow R by chloroperoxidase from Caldariomyces fumago: catalytic mechanism and degradation pathway. Ind Eng Chem Res.

[9] Chowdhury S, Balasubramanian R (2014). Graphene/semiconductor nanocomposites (GSNs) for heterogeneous photocatalytic decolorization of wastewaters contaminated with synthetic dyes: a review. Appl Catal B.

[10] Hu M, Yan X, Hu X, Zhang J, Feng R, Zhou M (2018). Ultra-high adsorption capacity of MgO/SiO_2_ composites with rough surfaces for Congo red removal from water. J Colloid Interface Sci.

[11] Munagapati VS, Yarramuthi V, Kim Y, Lee KM, Kim DS (2018). Removal of anionic dyes (Reactive black 5 and Congo red) from aqueous solutions using banana peel powder as an adsorbent. Ecotoxicol Environ Saf.

[12] Yaneva ZL, Georgieva NV (2012). Insights into Congo red adsorption on agro-industrial materials-spectral, equilibrium, kinetic, thermodynamic, dynamic and desorption studies. A Review. Int Rev Chem Eng.

[13] Alexander C, Andersson HS, Andersson LI, Ansell RJ, Kirsch N, Nicholls IA, O’Mahony J, Whitcombe MJ (2006). Molecular imprinting science and technology: a survey of the literature for the years up to and including 2003. J Mol Recognit.

[14] Tse Sum Bui B, Haupt K (2010). Molecularly imprinted polymers: synthetic receptors in bioanalysis. Anal Bioanal Chem.

[15] Haupt K, Linares AV, Bompart M, Tse Sum Bui B, Haupt K (2012). Molecularly imprinted polymers. Topics in current chemistry.

[16] Luo X, Zhan Y, Huang Y, Yang L, Tu X, Luo S (2011). Removal of water-soluble acid dyes from water environment using a novel magnetic molecularly imprinted polymer. J Hazard Mater.

[17] Cirillo G, Curcio M, Parisi OI, Puoci F, Iemma F, Spizzirri UG, Restuccia D, Picci N (2011). Molecularly imprinted polymers for the selective extraction of glycyrrhizic acid from liquorice roots. Food Chem.

[18] Puoci F, Garreffa C, Iemma F, Muzzalupo R, Spizzirri UG, Picci N (2005). Molecularly imprinted solid phase extraction for detection of Sudan I in food matrices. Food Chem.

[19] Ng SM, Narayanaswamy R (2009). Molecularly imprinted β-cyclodextrin polymer as potential optical receptor for the detection of organic compound. Sens Actuators, B.

[20] Singh DK, Mishra S (2010). Synthesis and characterization of Hg(II)-ion-imprinted polymer: kinetic and isotherm studies. Desalination.

[21] Hou H, Zhou R, Wu P, Wu L (2012). Removal of Congo red dye from aqueous solution with hydroxyapatite/chitosan composite. Chem Eng J.

[22] Mahapatra A, Mishra BG, Hota G (2013). Adsorptive removal of Congo red dye from wastewater by mixed iron oxide–alumina nanocomposites. Ceram Int.

[23] Harsini NN, Ansari M, Kazemipour M (2018). Synthesis of molecularly imprinted polymer on magnetic core-shell silica nanoparticles for recognition of congo red. Eurasian J Anal Chem.

[24] Poole CF, Lee Mike S (2012). Mass Spectrometry Handbook. Chromatographia.

[25] Mayor MG, González GP, Martínez RG, Hernando PF, Alegría JD (2017). Synthesis and characterization of a molecularly imprinted polymer for the determination of Spiramycin in sheep milk. Food Chem.

[26] Komiyama M, Takeuchi T, Mukawa T, Asanuma H (2003). Molecular iImprinting: from fundamentals to applications.

[27] Rachkov A, Minoura N (2000). Recognition of oxytocin and oxytocin-related peptides in aqueous media using a molecularly imprinted polymer synthesized by the epitope approach. J Chromatogr A.

[28] Zhang Y, Xie Z, Teng X, Fan J (2016). Synthesis of molecularly imprinted polymer nanoparticles for the fast and highly selective adsorption of sunset yellow. J Sep Sci.

[29] Asiabi H, Yamini Y, Seidi S, Ghahramanifard F (2016). Preparation and evaluation of a novel molecularly imprinted polymer coating for selective extraction of indomethacin from biological samples by electrochemically controlled in-tube solid phase microextraction. Anal Chim Acta.

[30] Li L, Li X, Duan H, Wang X, Luo C (2014). Removal of Congo red by magnetic mesoporous titanium dioxide-graphene oxide core-shell microspheres for water purification. Dalton Trans.

[31] Kyzas GZ, Bikiaris DN, Lazaridis NK (2009). Selective separation of basic and reactive dyes by molecularly imprinted polymers (MIPs). Chem Eng J.

[32] Arabzadeh N, Abdouss M (2010). Synthesis and characterization of molecularly imprinted polymers for selective solid-phase extraction of pseudoephedrine. Colloid J.

[33] Madikizela LM, Zunngu SS, Mlunguza NY, Tavengwa NT, Mdluli PS, Chimuka L (2018). Application of molecularly imprinted polymer designed for the selective extraction of Ketoprofen from wastewater. Water SA.

[34] Nantasenamat C, Naenna T, Ayudhya CIN, Prachayasittikul V (2005). Quantitative prediction of imprinting factor of molecularly imprinted polymers by artificial neural network. J Comput Aided Mol Des.

[35] Dai CM, Geissen SU, Zhang YL, Zhang YJ, Zhou XF (2011). Selective removal of diclofenac from contaminated water using molecularly imprinted polymer microspheres. Environ Pollut.

[36] Azodi-Deilamia S, Abdoussa M, Seyedib SR (2010). Synthesis and characterization of molecularly imprinted polymer for controlled release of tramadol. Open Chemistry.

[37] Bashir S, Teo YY, Ramesh S, Ramesh K (2016). Synthesis, characterization, properties of N-succinyl chitosan-g-poly (methacrylic acid) hydrogels and in vitro release of theophylline. Polymer.

[38] Li J, Ng DH, Song P, Kong C, Song Yang P (2015). Preparation and characterization of high-surface-area activated carbon fibers from silkworm cocoon waste for Congo red adsorption. Biomass Bioenerg.

[39] Minhas MU, Ahmad M, Ali L, Sohail M (2013). Synthesis of chemically cross-linked polyvinyl alcohol-co-poly (methacrylic acid) hydrogels by copolymerization; a potential graft-polymeric carrier for oral delivery of 5-fluorouracil. DARU J Pharm Sci.

[40] Azhgozhinova GS, Güven O, Pekel N, Dubolazov AV, Mun GA, Nurkeeva ZS (2004). Complex formation of linear poly (methacrylic acid) with uranyl ions in aqueous solutions. J Colloid Interface Sci.

[41] Tamayo FG, Casillas JL, Martin-Esteban A (2005). Evaluation of new selective molecularly imprinted polymers prepared by precipitation polymerization for the extraction of phenylurea herbicides. J Chromatogr A.

[42] Roland RM, Bhawani SA (2016). Synthesis and characterization of molecular imprinting polymer microspheres of piperine: extraction of piperine from spiked urine. J Anal Methods Chem.

[43] Chow J, Leong A, Bhawani SA (2016). Synthesis and characterization of molecular imprinting polymer microspheres of cinnamic acid: extraction of cinnamic acid from spiked blood plasma. Int J Pol Sci..

[44] Bhawani SA, Sen TS, Ibrahim MN (2018). Synthesis of molecular imprinting polymers for extraction of gallic acid from urine. BMC Chem.

[45] Mane S, Ponrathnam S, Chavan N (2015). Design and synthesis of cauliflower-shaped hydroxyl functionalized core-shell polymer. Des Monomers Polym.

[46] Esfandyari-Manesh M, Javanbakht M, Atyabi F, Badiei A, Dinarvand R (2011). Effect of porogenic solvent on the morphology, recognition and release properties of carbamazepine-molecularly imprinted polymer nanospheres. J Appl Polym Sci.

[47] Farzi G, Mortezaei M, Badiei A (2011). Relationship between droplet size and fluid flow characteristics in mini emulsion polymerization of methyl methacrylate. J Appl Polym Sci.

[48] Luo X, Zhan Y, Huang Y, Yang L, Tu X, Luo S (2011). Removal of water-soluble acid dyes from water environment using a novel magnetic molecularly imprinted polymer. J Hazard Mater.

[49] Asman S, Mohamad S, Sarih NM (2015). Influence of polymer morphology on the adsorption behaviors of molecularly imprinted polymer-methacrylic acid functionalized β-cyclodextrin. J Appl Polym Sci.

[50] Song R, Hu X, Guan P, Li J, Qian L, Wang C, Wang Q (2015). Synthesis of porous molecularly imprinted polymers for selective adsorption of glutathione. Appl Surf Sci.

